# Intimate partner violence among Nepalese women and associated factors in 2021–2022

**DOI:** 10.1371/journal.pgph.0003267

**Published:** 2025-03-25

**Authors:** Masood Ali Shaikh

**Affiliations:** Independent Researcher, Karachi, Pakistan; University of Washington Department of Global Health, UNITED STATES OF AMERICA

## Abstract

Intimate partner violence (IPV) remains a significant public health challenge globally, impacting millions and contributing to preventable suffering and pain, both emotionally and physically. In Nepal, like many other nations, tackling IPV is a key priority on the path to achieving sustainable development goals. IPV prevalence and its correlates were studied using the deidentified data for secondary analysis from the 2022 Nepal Demographic and Health Survey (DHS), and changes in IPV at the urban, rural, and national levels from the 2016 Nepal DHS. These cross-sectional surveys employed a two-stage cluster sampling technique. In the Nepal DHS 2022, a total of 4,523 ever-partnered women were included and administered IPV-related questions. Various socio-demographic, attitudinal, and experiential attributes of 15-49 year old women were identified and used in the simple and multiple logistic regression models for computing the bivariate and multivariable associations with IPV. Over their lifetime, 27.27% women experienced IPV perpetrated by their current or most recent husband/intimate partner, with physical IPV being the most common type reported. The six statistically significant factors associated with IPV in the multivariable model included educational level of women and their partner, number of living children, use of alcohol by partner, knowing about father having ever beaten mother, and controlling behavior displayed by partner. While changes in IPV from 2016 to 2022 were not statistically significant. One in four women having experienced IPV in their lifetime in Nepal with no discernable change over the course of 6-years is a major public health challenge requiring investing in programs that encourage and ensure economic and social autonomy for women and change in the harmful gender norms.

## Introduction

Intimate partner violence (IPV) against women is an enduring and endemic public health scourge and a human rights injustice through millennia. It entails emotional, physical, and/or sexual violence and formally defined by the World Health Organization (WHO) as “*any behavior within an intimate relationship that causes physical, psychological, or sexual harm to those in the relationship*” [1]. To address this scourge, the Sustainable Development goal 5 – target 5.2 of the United Nations envisions to “*Eliminate all forms of violence against all women and girls in the public and private spheres, including trafficking and sexual and other types of exploitation*” [2].

In 2018, the global lifetime prevalence estimate of physical and/or sexual IPV for ever-married/ partnered women aged 15-49 was 27% [3]. However, in the WHO South-East Asia region that includes Nepal, the lifetime prevalence was 33%. The Demographic and Health Surveys (DHS) are conducted by the U.S. Agency for International Development (USAID) through its ‘Demographic and Health Surveys Program’. DHSs are nationally and sub-nationally representative and for many developing countries, these are the only surveys that provide reliable and representative indices on IPV. These surveys have been conducted in over 90 countries using standardized methodology [4].

DHS data, a multifaceted lens, has been instrumental in examining the true scope and intricate relationships between IPV prevalence and complex web of correlates. Studies using data from the cross-sectional DHS and other surveys have reported a surfeit of IPV correlates that include demographic and socio-economic factors pertaining to women and their intimate partners. These include age and educational attainment of women and their intimate partner, women’s employment status, residency status, household wealth, exercise of male partners controlling behaviors and alcohol use, knowledge of father having ever beaten mother, participation in decision-making and acceptance of IPV by women[5–25]. Additionally, several meta analyses have been reported providing combined results from several countries on the correlates of IPV in developing countries. Two separate meta-analyses, based on DHSs data conducted in sub-Saharan African countries have reported on IPV’s associations: one study identified poor living conditions, rural residency status, and low educational attainment in women as correlates of IPV from 25 countries [26], while another study reported IPVs association with low educational attainment in women using data from 29 countries [27]. Another meta-analysis based on 25 studies from low and middle income countries reported IPVs association with infertility in women [28]. While association of unplanned pregnancy as a high risk for IPV and older age bestowing protection against IPV was reported in a study using 36 prospective studies [26]. While higher prevalence of IPV was reported in infertile women in a meta-analysis using 25 studies from low- and middle-income countries [28]. A study based on 36 prospective studies reported unplanned pregnancy and low parental educational attainment (less than high school) as IPV risk factors, while being married and of older age as shielding against IPV [29].

Nestled amidst the Himalayas, the south Asian country of the Federal democratic Republic of Nepal battles the high burden of IPV. Based on results from the 2016 DHS conducted in Nepal, 26.33% of ever-married women reported having ever experienced emotional, physical, and/or sexual IPV perpetrated by their husbands [30].

Several studies have reported on the IPV against women in Nepal. A study based on 1,800 women from three districts in Nepal, reported 25.28% physical and/or sexual IPV prevalence and identified several statistically significant correlates in a multivariable model including frequent drunkenness of husband, experiencing financial stress, women earning less or equivalent to her husband, and having witnessed IPV as a child by women and her husband [31]. Comparing lifetime physical and/or sexual IPV prevalence using data from the 2016 and 2011 DHSs in Nepal, a statistically significant decline was reported over the course of two surveys [32]. A recent study based on web-based survey reported that during the COVID-19, 52.62% of 420 married women experienced IPV [33]. Another study based on a sample of 602 married women from one municipality of Kathmandu district in Nepal, reported an IPV prevalence of 22.1% [34].

This study aimed to examine the prevalence and correlates of IPV in Nepal using the most recent, i.e., 2022, nationally and sub-nationally representative DHS cross-sectional survey data for secondary analysis. The potential findings of this study are useful for Nepal’s journey towards the realization of the Sustainable Development Goal 5, which seeks to achieve gender equality and empower all women and girls by 2030, and also in formulating policies and interventions that will reduce IPV prevalence in Nepal.

## Methods

### Ethics Statement

For Nepal Demographic and Health Survey 2022, the ethical review and approval was provided by the “Suaahara II” which is a home-based nutritional program for women and children in Nepal, supported by the USAID in collaboration with the Nepali government. While the Measure DHS granted approval for the secondary analysis reported in this study to the author and provided fully deidentified data files for NDHS2016 and NDHS2022.

### Study area and data source

Fully deidentified data from the cross-sectional Nepal 2016 (NDHS2014) and 2022 (NDHS2022) Demographic and Health Surveys were used in this study for secondary analysis. The NDHS2021-22 was conducted by the ‘New ERA’ which is the Nepal’s first non-governmental non-profit research organization, supported by the Nepal Ministry of Health and Population. While technical assistance was provided by the ICF through the DHS Program.

The NDHS2021-22 data collection period lasted from January 5 to June 22, 2022. The updated 2011 Nepal Population and Housing Census was used as a sampling frame for the NDHS2022. A two-stage stratification was used for sample selection: in the first stage, using probability proportional to size sampling, 476 primary sampling units (PSUs) were selected. In the second stage, all households within each PSU were listed and 30 households were selected from each PSU: resulting in the selection of 14,280 households cumulatively. From these households 7,140 were selected for the men’s questionnaire, and a subsample of these households were subsequently selected for the ‘domestic violence’ (DV) questionnaire that included IPV questions. Using World Health Organization’s guidelines for the ethical collection of information pertaining to domestic violence and IPV, one woman from each household was selected and interviewed [35]. In order to ensure that this selection methodology was nationally representative, special weights were generated and used in the analysis. Cumulatively, 5,192 women 15-49 year old who were either permanent residents or had stayed overnight in the chosen households, were selected for the administration of DV questionnaire that included IPV questions. But 14 selected women either could not be contacted or privacy during interview could not be ensured, thus resulting in the administration of DV module to 5,178 women. However, 4,523 women were asked IPV questions for NDHS2022, as the rest did not report having ever had an intimate partner. Similarly for NDHS2016, 3,826 women were successfully asked IPV questions. For NDHS2016 IPV questions were only asked from those women who were ever married, i.e., women who had either a current/former husband.. However, for NDHS2022, the eligibility criteria was broadened to also include women who were never married but either had a current or a former intimate partner. Thus for NDHS2022 IPV questions were asked from all women who ever had a current/former husband or current/former intimate partner. Lifetime IPV prevalence and trend analysis was done using the new expanded inclusion criteria for the NDHS2022.

Complete survey methodology including sampling methodology, generation of survey weights for the DV module were published in the Nepal DHS country reports. These reports are available for free download on the DHS website at www.measuredhs.com.

### Study variables

In Nepal, as in other countries, the DHS employed a standardized domestic violence module based on a modified Conflict Tactics Scale (CTS). This modified CTS has been empirically validated and demonstrated good reliability in both community and clinical settings [36–37]. The module uses specific questions to assess emotional, physical, and sexual violence perpetrated by the male intimate partner. The computation of outcome and explanatory variables based on this data is described in detail below.

### Outcome variable

Intimate partner violence (IPV) was assessed as a dichotomous (Yes/No) variable, coded as 1 if the respondent reported experiencing any type of emotional, physical, or sexual violence from her current or most recent male husband/intimate partner at any point in her life. The term “husband/intimate partner” encompasses the following definitions: for women who were currently married, it refered to their current male husband; for those who were divorced, separated, or widowed, it refered to their most recent husband; for never-married women with a current male intimate partner, it refered to that partner; and for never-married women without a current partner but with a past intimate relationship, it refered to their most recent intimate partner.

A respondent was considered to have experienced emotional IPV if she affirmed ever experiencing being made to feel bad, threatened to be harmed, insulted, or humiliated by her male husband/intimate partner. Physical IPV was based on respondent’s affirmative responses to either having been ever pushed, shaken, thrown something at, slapped, arm twisted or hair pulled, punched with fist or something that could hurt, kicked, dragged, strangled, burned, or threated with a knife, gun, or any weapon by her husband/male intimate partner. While sexual violence was computed as respondent ever having been either physically forced into unwanted sex, unwanted sexual acts, or to perform unwanted sexual acts.

### Explanatory variables

Previous studies using DHS data have identified factors associated with IPV. This study investigated twelve potential associations: Demographic characteristics: women’s age and occupation, education level of both women and husband/partners, and women’s occupation. Socioeconomic factors: household wealth quantile, urban/rural residence, number of living children. Women’s empowerment: Ability to participate in decision-making (healthcare, major purchases, visiting family/friends) in terms of either respondent decides alone or jointly with husband/intimate partner. Acceptance of IPV: Respondent agrees with statements justifying wife beating under certain specific circumstances (refusing sex, arguing, neglecting children, burning food, and/or going out without telling her husband). Exposure to violence: knowledge of father having ever beaten mother. Husband/intimate partner’s controlling behavior; Respondent affirms experiencing any of the following: jealous or angry if she talks to other men, accusations of infidelity, restricting social interactions by limiting contact with her family, not permitting her to meet women friends, or demanding constant whereabouts information. Finally, use of alcohol by her husband/intimate partner.

### Statistical analysis

All analyses were performed in STATA version 18 (Texas, USA) factoring in the complex survey design; P value of less than 5% (<0.05) was used to designate statistical significance. Nepal DHSs deidentified datasets in STATA file format were downloaded from the DHS website. Descriptive analyses were used to describe attributes and outcomes. Descriptive statistics, including unweighted counts, number of missing values, and weighted proportions, were calculated for all variables without imputation. Binary logistic regression models were then built for each explanatory variable to predict the probability of the binary IPV outcome. Only those variables that were statistically significant at the level of <0.10 from the individual models were kept for the final multiple logistic regression analysis. For each, odds ratios, their 95% confidence intervals, and statistical significance levels are reported. Multicollinearity among all the explanatory variables was assessed, and the Goodness-of-Fit test for the final multivariable model was conducted to verify its adequacy. Focusing on temporal changes in IPV prevalence, linear trend analyses were conducted for each type of IPV (emotional, physical, and sexual) and for their combined prevalence. These analyses were performed at both national and urban/rural levels using data from the NDHS16 and NDHS2022 surveys. To achieve this, the two survey datasets were combined, and a new dichotomous variable “year” was created to indicate participation in either survey. This “year” variable served as the dependent variable in the logistic regression models, with the different IPV types as the explanatory variable.

## Results

In total 1,178 (27.27%) women reported having ever experienced emotional, physical, and/or sexual IPV perpetrated by their current or most recent husband/intimate partner. Regarding each type of violence ever experienced: 563 (13.41%) women reported emotional IPV, 992 (23.13%) women reported physical IPV, and 323 (7.28%) women reported sexual IPV.

Concerning the frequency of combinations of different forms of intimate partner violence (IPV) ever experienced: emotional, sexual, and physical, or all three types, 177 women (4.28%), physical and emotional IPV by 417 women (9.85%), physical and sexual IPV by 266 women (6.41%), and 194 women (4.57%) reported experiencing emotional and sexual IPV. While 1,049 women (23.99%) reported ever experiencing sexual and/or physical IPV.

Table 1 shows the number (unweighted) and percentage (weighted) of women who experienced intimate partner violence (IPV) and its different types. It also shows information about the twelve factors considered in this analysis, including the number of women with missing data for each factor.

**Table 1:** Counts and proportions of study variables – Nepal Demographic and Health Survey 2022.

Variable	Unweighted count	Percentage
	(N = 4,523)	(Weighted)
Outcome Variable		
Intimate Partner Violence		
(Emotional, physical, and/or sexual)	Yes = 1,178	27.27 (25.37–29.26)
Emotional violence	Yes = 563	13.41 (11.75–15.26)
Physical violence	Yes = 992	23.13 (21.36–25.00)
Sexual violence	Yes = 323	7.28 (6.29–8.41)
Explanatory Variables		
Age	15–19y = 243	6.96%
	20–24y = 709	16.86%
	25–29y = 903	18.94%
	30–34y = 869	16.90%
	35–39y = 741	15.79%
	40–44y = 580	13.40%
	45–49y = 478	11.15%
Respondent’s Education	No education = 1,429	30.97%
	Primary =1,552	32.31%
	Secondary = 1,411	32.99%
	Higher = 131	3.73%
Husband/Partner’s Education	No education/Don’t know = 618	15.84%
	Primary = 1,77239.95%	
	Secondary = 1,569	37.55%
	Higher = 252	6.66%
	Not Applicable = 312	
Occupation	Professional/Technical/Managerial/Clerical/Sales = 604	15.87%
	Not working = 828	23.53%
	Agriculture: self-employed/Skilled manual/Unskilled manual/Other = 3,091	60.60%
Wealth	Poorest = 1,321	18.65%
	Poorer = 918	19.15%
	Middle = 886	20.75%
	Richer = 812	21.77%
	Richest = 586	19.69%
Residence	Urban = 2,371	67.39%
	Rural = 2,152	32.61%
Children	0 = 476	14.07%
	1–2 = 2,565	55.43%
	3–4 = 1,268	25.77%
	5–10 = 214	4.73%
Decision making	Participated = 3,624	83.74%
	Not participated = 587	16.26%
	Not Applicable = 312	
Acceptance	Not justified/Don’t know = 3,651	80.88%
	Justified = 872	19.12%
Alcohol use by partner/husband	No = 2,181	48.75%
	Yes = 2,342	51.25%
Knowledge of Parental IPV	No/Don’t know = 3,775	82.77%
	Yes = 748	17.23%
Controlling behavior	No = 3,022	64.90%
	Yes = 1,501	35.10%

* Questions on ‘Husband/partner’s education’ and questions pertaining to ‘Decision making’ were asked from only those women who were either currently married or currently living with a male intimate partner.

While 312 women were not asked questions about “Husband/partner’s education” and involvement in “Decision making,” this was intentional to ensure the collected data specifically reflected partnered dynamics for women who were either currently married or currently living with a man in an intimate relationship.

While all three types of IPV were measured by composite indicators, ‘ever being insulted or made to feel bad’ by an intimate partner stood out as the most common form of emotional abuse, affecting 10.18% of women; 20.67% women reported ‘Ever been slapped’ by a husband/intimate partner, and it was the most commonly reported physical abuse; while being ‘physically forced into unwanted sex’ by husband/intimate partner was the commonest (6.87%) form of sexual abuse reported.

The respondent profile showed that 40.34% of women were between the ages of 35 and 49; 30.97% of women and 15.84% of their husbands/intimate partners were illiterate; 23.53% of women were unemployed; 41.46% of women belonged to the wealthiest (richer or richest) quantile; 32.61% of women lived in rural areas; 55.43% of women had one or more living children; 83.74% of women reported participating in decision-making; and 80.88% of women thought that IPV was not justified; Only 17.23% of women reported knowing that their father had ever physically beaten their mother, 51.25% of women indicated that their husband/intimate partner used alcohol, and 35.10% of women reported that their husband/intimate partner showed controlling behavior.

Table 2 displays the findings of both simple and multivariable logistic regression models as crude and adjusted odds ratios, along with the related 95% confidence intervals and statistical significance. Out of the twelve explanatory variables examined, only one—the residence status (urban or rural) —was found to be not statistically significantly related to the experience of IPV in the simple logistic regression model. Hence, the multivariable logistic regression model included the remaining eleven covariates: the women’s age, level of education attained by the woman and her husband/intimate partner, their financial situation, the number of children who are alive, the occupation of the woman, whether or not she accepts intimate partner violence, her participation in decision-making, whether or not their husband/partner uses alcohol, knowing of father having ever beaten mother, and the controlling behavior that the husband/intimate partner exhibits. With the exception of women’s age, employment status, household wealth, participation in decision-making, and her acceptance of IPV, all remaining six factors in the final multivariable logistic regression model were statistically significantly linked to the experience of IPV by women.

**Table 2:** Crude odds ratios and adjusted odds ratios for all statistically significant associations between intimate partner violence and the selected variables – Nepal Demographic and Health Survey 2022.

Explanatory variable	Unadjusted OR	P value	CI	Unadjusted OR	P value	CI
Age						
15–19	Reference			Reference		
20–24	1.06	0.825	0.64–1.74	0.76	0.419	0.40–1.47
25–29	1.29	0.285	0.81–2.05	0.81	0.484	0.44–1.48
30–34	1.65	0.032	1.04–2.62	1.05	0.878	0.57–1.93
35–39	1.39	0.167	0.87–2.22	0.79	0.465	0.42–1.49
40–44	1.62	0.037	1.03–2.56	0.76	0.411	0.40–1.45
45–49	1.38	0.189	0.85–2.23	0.69	0.273	0.36–1.34
Education (Respondent/Women)						
No Education	Reference			Reference		
Primary	0.61	<0.001	0.51–0.73	0.63	0.001	0.48–0.83
Secondary	0.33	<0.001	0.27–0.41	0.48	<0.001	0.34–0.69
Higher	0.09	<0.001	0.04–0.18	0.19	<0.001	0.08–0.45
Education (husband/partner)						
No Education	Reference			Reference		
Primary	0.61	<0.001	0.49–0.77	0.73	0.015	0.56–0.94
Secondary	0.33	<0.001	0.26–0.43	0.66	0.012	0.47–0.91
Higher	0.13	<0.001	0.08–0.22	0.50	0.027	0.27–0.92
Occupation						
Professional, Technical, Managerial, Clerical, Sales	Reference			Reference		
Not working	1.68	0.003	1.19–2.36	1.12	0.565	0.76–1.66
Agriculture, Self-employed, Skilled manual, Unskilled manual, Other	2.02	<0.001	1.52–2.68	1.08	0.660	0.77–1.52
Wealth						
Poorest	Reference			Reference		
Poorer	1.38	0.009	1.08–1.77	1.36	0.032	1.03–1.81
Middle	1.16	0251	0.90–1.51	1.21	0.231	0.89–1.64
Richer	0.99	0.916	0.76-1.28	1.33	0.084	0.96–1.83
Richest	0.51	<0.001	0.38–0.69	1.11	0.612	0.74–1.66
Residence		0.009	1.08–1.77	1.36	0.032	1.03–1.81
Urban	Reference			Not Applicable		
Rural	1.03	0.781	0.84–1.27			
Children						
No children	Reference			Reference		
1–2 children	1.55	0.010	1.11–2.17	1.29	0.283	0.81–2.05
3–4 children	2.78	<0.001	2.01–3.83	2.00	0.006	1.22–3.27
5–10 children	3.21	<0.001	1.99–5.17	2.08	0.039	1.04–4.16
Decision making						
Did not participate	Reference			Reference		
Participated	0.67	0.002	0.52–0.86	0.80	0.126	0.60–1.07
Acceptance of IPV						
Not justified	Reference			Reference		
Justified	1.39	0.003	1.12–1.72	1.18	0.188	0.92–1.50
Alcohol use by partner/husband						
Does not use alcohol	Reference			Reference		
Uses alcohol	Reference	<0.001	1.92–2.65	1.76	<0.001	1.45–2.13
Knowledge of Parental IPV						
No	Reference			Reference		
Yes	2.47	<0.001	2.01–3.03	2.06	<0.001	1.60–2.64
Controling behavior						
No	Reference			Reference		
Yes	6.09	<0.001	5.08–7.30	6.25	<0.001	5.15–7.58

OR = Odds Ratio. CI = Confidence Interval.

Table 2’s results from the multivariable logistic regression model indicate the following statistically significant associations: women with either primary (aOR: 0.63; 95% CI: 0.48-0.83), secondary (aOR: 0.48; 95%CI: 0.34-0.69), or higher (aOR: 0.19; 95%CI: 0.08-0.45) educational level were protected against IPV compared to women with no education. Comparably, women were shielded against IPV by husbands/intimate partners who had either primary (aOR: 0.73; 95% CI: 0.56-0.94), secondary (aOR: 0.66; 95%CI: 0.47-0.91) or higher (aOR: 0.50; 95%CI: 0.27-0.92) educational attainment compared to women with husbands/intimate partners with no education. Women who had 3-4 children (aOR: 2.00; 95%CI: 1.22-3.27), or women with 5 or more children (aOR: 2.08; 95%CI: 1.04-4.16) were more likely to have ever experienced intimate partner violence (IPV) than women who had no children. Women in poorer wealth quantile (aOR: 1.36; 95%CI: 1.03-1.81) were more likely to experience IPV compared with women in the poorest wealth quantile. Women who had alcohol-using husbands/intimate partners were more likely to report IPV (aOR: 1.76; 95%CI: 1.45-2.13) than women whose husbands/partners did not use alcohol. When compared to women who were unaware or had no knowledge, knowledge of one’s mother having ever been beaten by one’s father was linked to increased (aOR: 2.06; 95%CI: 1.60-2.64) odds of having ever experienced IPV. Lastly, compared to women whose husband/intimate partner did not exhibit controlling conduct, those whose husband/intimate partner did, had a higher likelihood (aOR: 6.25; 95%CI: 5.15-7.58) of reporting having ever experienced IPV. The multivariable model pertains to women who were either currently married or currently living with a men in the context of intimate relationship, as this model included factors of their husband’s/partner’s education and their involvement in decision-making.

Multicollinearity between all the explanatory variables in the final multivariable logistic model was determined by the Variance Inflation Factor (VIF) and was less than 2.11 in all the variables. The results of the goodness-of-fit test indicate that the multivariable logistic regression model for the IPV was satisfactory ([F(9,454]) = 0.27; p-value: 0.9817.

Table 3 displays the findings of a linear trend analysis comparing the three categories and cumulative IPV at the national, rural, and urban levels between the NDHS2016 and the NDHS2022. Albeit in general, there was a slight uptick cumulatively as well as in all three forms of IPV across urban, rural, and national levels in 2022, none of the trends were determined to be statistically significant.

**Table 3:** Proportions and trend analyses of emotional, physical, sexual, and cumulative intimate partner violence (IPV) by urban/rural residency status - Nepal Demographic and Health Surveys (DHSs) 2016 and 2021–22.

Type of violence	DHS 2016		DHS 2021-22		Trend Statistical Significance
	Percentage	95% CI	Percentage	95% CI	(p-value)
Cumulative IPV					
National	26.33	24.37–28.39	27.27	25.37–29.26	0.508
Urban	25.41	22.98–28.01	27.08	24.76–29.54	0.344
Rural	27.70	24.49–31.16	27.67	24.42–31.16	0.988
Emotional Violence					
National	12.30	10.85–13.91	13.41	11.75–15.26	0.346
Urban	11.74	9.86–13.93	13.51	11.50–15.79	0.241
Rural	13.12	10.94–15.67	13.21	10.41–16.63	0.964
Physical Violence					
National	22.80	20.87–24.85	23.13	21.36–25.00	0.811
Urban	21.98	19.62–24.52	23.11	20.89–25.49	0.507
Rural	24.04	20.84–27.55	23.17	20.38–26.23	0.702
Sexual Violence					
National	7.05	6.00–8.26	7.28	6.29–8.41	0.773
Urban	7.08	5.87–8.52	7.26	5.97–8.82	0.852
Rural	7.00	5.23–9.31	7.30	6.02–8.83	0.811

CI = Confidence Interval.

## Discussion

Intimacy and violence may seem contradictory, but it is a global opprobrium. IPV remains a persistent scourge in Nepal, where its shadow continues to loom large, with about one in four women experiencing physical, sexual, or emotional abuse in their lifetime perpetrated by their current or most recent male husband/intimate partner. This study analyzed the prevalence and associated factors of intimate partner violence in Nepal using the 2022 National Demographic and Health Survey (NDHS) and compares trends across national, urban, and rural areas based on the 2016 NDHS data. Based on the results presented here, using the most recent representative survey, 27.27% of Nepalese women had experienced some form of IPV in their lifetime. Against the backdrop of 27% lifetime prevalence of physical and/or sexual IPV reported globally, in Nepal lifetime prevalence of these two IPV types was 24% [3].

Physical IPV was the most common form reported by Nepalese women, consistent with findings from other countries using DHS data [13,16,18,22]. Situations where physical violence is more likely to occur might be influenced by societal norms and expectations surrounding gender roles. A culture of violence that impacts women in intimate relationships can be attributed to societal issues such as toxic masculinity and gender stereotypes. This grim situation underscores the critical need for both effective health promotion programs and the integration of gender equality into society.

In the bivariate analyses elucidating IPV’s association with the twelve factors studied, only urban/rural residency status was not statistically significant. However evidence for residency status is equivocal, as higher association of rural residency and IPV [17,27], lower association of rural residency with IPV^12^, as well as no association of residency status with IPV [18,22], have been reported. Lack of statistical association of residency status and IPV could imply more generalized acceptance of IPV that is independent of residency status.

In the multivariable model, examining associations of IPV with the remaining eleven factors, women’s age, occupation, household’s wealth, participation in decision-making, and acceptance of IPV, were not statistically significant. Association of older age with higher IPV [13], protective effect of older age with IPV [29], association of younger age with higher IPV [10], and no association of age with IPV [18,22], have all been reported. Lack of association in this study, between IPV and age might be construed as widespread occurrence of IPV in the country. Evidence of women’s occupation and IPV association is mixed as well, unemployment being protective against IPV [7], no association between the two [18,22], and her employment, notably if she is the only one who worked, or women who earn more than their partner are reported to be associated with higher IPV [11]. Women’s role and involvement in decision-making pertinent to important issues – either personal or family – and its association with IPV has been reported to be protective against IPV [14] or not statistically significant [18,22]. Lack of statistically significant association in this study perhaps points towards pervasiveness of IPV, congruent with its acceptance in the context of intimacy. Regarding acceptance of IPV by women and its association with IPV; association of higher IPV in women who report its acceptance has been reported [12,13,22], while no such statistically significant association has also been reported [18]. Whether IPV acceptance precede and predict IPV is daunting to discern in the context of a cross-sectional study like DHS. However, the possibility of reverse causation phenomenon [38] experiencing IPV preceding its acceptance, could not be ruled out either.

In the multivariable model, seven factors were found to be statistically significantly associated with IPV, that included, educational level of women and their husband/intimate partner, number of living children, wealth quantile, use of alcohol by partner, knowing about father having ever beaten mother, and controlling behavior displayed by husband/intimate partner. Educational attainment, especially higher level/years of education attained by women and their husbands/intimate partners has been fairly consistently associated with lower levels of IPV in women [11,13,18]. On the other hand, low educational attainment’s association with higher levels of IPV in women [10,27], and in their partner has been reported [7,10,14]. Education perhaps confers better sense of fairness and nonviolent ways of handling stress in the context of intimate relationships. Having higher number of children and its association with IPV has also been fairly consistently reported empirically [7,10,12,17,22]. Whereas association of infertility in women and higher prevalence of IPV has similarly been reported [28], in addition to no association between having children and IPV [18]. Perhaps stress of raising and providing for children serves as a trigger for IPV in some men. Preponderance of evidence indicates that higher household wealth status is associated with lower IPV in women, compared with women in lower economic strata [8,9,11,17,18], conversely lower wealth status with higher IPV has been reported [19]; however, no association between the two has also been reported [22]. Wealth perhaps insulates from the stress of providing for and financially taking care of one’s family by men and the resultant morbid indulgence in IPV. Association of alcohol use by the husband/intimate partner and IPV has been rather well established [7,8,10,12,14–16,20,22]. Alcohol use impacts judgement and reduces inhibitions and perhaps contributes of IPV [25]. Knowledge of father having ever beaten mother is a distressing experience and its association with IPV has also been widely and consistently reported [5,7,8,10,12,13,16,18,21–23]. Perhaps such knowledge reinforces acceptance of IPV by some women against the backdrop of IPV’s social acceptance. However, equally plausible is the fact of women being more likely to recall such knowledge when they themselves have been subjected to IPV in their own intimate relationships. Finally, controlling behavior shown by husband/intimate partner and its association with higher IPV is another consistent finding reported [8,12,16,18,24]. Controlling behavior reflects pathological possessiveness and manifests as violence in the context of intimate relationships. However, results of multivariable model are applicable to women who were either currently married or currently living with a man in the context of an intimate relationship.

Regarding change in IPV, cumulatively, as well as its three types in Nepal, over the course of two DHSs conducted in 2016 and 2022 exhibited no statistically significant change in either direction at either national, urban, or rural levels. Despite the broadened definition of male intimate partner used in 2022. Albeit there was a slight increase in almost all IPV indices at three geographical levels from 2016 to 2022. Perhaps the interval of five years between the two surveys was not long enough to display statistically significant change, but nonetheless augurs for the need to better address this health and human rights issue in the country. Previous studies from other countries have shown statistically significant differences in IPV types based on subnational administrative/geographic subdivisions during the similar time intervals [18,39,40]. However, gender bias remains a deeply entrenched issue across the globe. Data from the 2023 United Nations Development Program’s “Gender Social Norms Index” (GSNI), based on information up to 2022, paints a concerning picture [41]. The report reveals that a staggering 85% of the world’s population harbors gender biases, highlighting their pervasiveness regardless of regional, economic, or cultural contexts. This translates to an alarming nine out of every ten people, both men and women, holding discriminatory views against women. Even more distressing is the finding that over 25% of respondents consider wife beating by a husband to be justified.

While this study utilized data from a robust, nationally representative survey with standardized tools, its cross-sectional design poses inherent limitations. The study’s reliance on a cross-sectional design inherently hinders its ability to draw causal conclusions. Firstly, it can only establish associations, not causal relationships between IPV and its correlates. Secondly, its exclusive focus on heterosexual partnerships excludes women in same-sex relationships experiencing IPV, potentially obscuring the full scope of the issue and underestimating the overall burden. Thirdly, the design cannot rule out ‘reverse causality,’ where past IPV experiences might influence attitudes towards it or recollections of parental violence. Fourthly, DHSs relying solely on self-reported data are vulnerable to underestimation, as shame and social stigma can discourage accurate disclosure. Finally, the expansion of the inclusion criteria for determining IPV, from ever-married women in 2016 to never-married women with intimate relationships in 2022 might have influenced the change in IPV burden over the course of two surveys.

Conducting longitudinal studies offers a powerful tool to shed light on the causal mechanisms and key drivers of IPV, moving beyond the limitations of cross-sectional surveys. Investing in programs that simultaneously unlock economic and social autonomy for women, challenge harmful gender norms, and equip couples with healthy relationship tools, provides a compelling roadmap towards a future free from intimate partner violence in Nepal.

## Conclusion

Using the most recent DHS data, one in four women reported having ever experienced some form of intimate partner violence in Nepal, with physical violence being the most common form reported. As in several other studies, association of education with lower reporting of IPV, and higher IPV in women reporting alcohol use by intimate partner was borne out. While knowing about father’s perpetration of physical violence against mother and controlling behavior exerted by partner were the other two strong correlates of IPV reinforced in this study. However, cross-sectional study design obviates causal relationships. Trends gleaned from this analysis suggest stagnancy in IPV indices at the urban, rural, and national levels. Reducing IPV burden and ultimately its prevention for a better, more inclusive, and fair future for women would involve capitalizing in programs that encourage and ensure economic and social autonomy for women and change in the harmful gender norms.
